# Neuropilin-2 Mediated β-Catenin Signaling and Survival in Human Gastro-Intestinal Cancer Cell Lines

**DOI:** 10.1371/journal.pone.0023208

**Published:** 2011-10-20

**Authors:** Shaija Samuel, Puja Gaur, Fan Fan, Ling Xia, Michael J. Gray, Nikolaos A. Dallas, Debashish Bose, Cristian Rodriguez-Aguayo, Gabriel Lopez-Berestein, Greg Plowman, Anil Bagri, Anil K. Sood, Lee M. Ellis

**Affiliations:** 1 Department of Cancer Biology, The University of Texas M. D. Anderson Cancer Center, Houston, Texas, United States of America; 2 Department of Surgical Oncology, The University of Texas M. D. Anderson Cancer Center, Houston, Texas, United States of America; 3 Department of Gynecologic Oncology, The University of Texas M. D. Anderson Cancer Center, Houston, Texas, United States of America; 4 Department of Experimental Therapeutics, The University of Texas M. D. Anderson Cancer Center, Houston, Texas, United States of America; 5 Center for RNA Interference and Non-Coding RNA, The University of Texas M. D. Anderson Cancer Center, Houston, Texas, United States of America; 6 Tumor Biology and Angiogenesis, Genentech, Inc., South San Francisco, California, United States of America; The University of Hong Kong, China

## Abstract

NRP-2 is a high-affinity kinase-deficient receptor for ligands belonging to the class 3 semaphorin and vascular endothelial growth factor families. NRP-2 has been detected on the surface of several types of human cancer cells, but its expression and function in gastrointestinal (GI) cancer cells remains to be determined. We sought to determine the function of NRP-2 in mediating downstream signals regulating the growth and survival of human gastrointestinal cancer cells. In human gastric cancer specimens, NRP-2 expression was detected in tumor tissues but not in adjacent normal mucosa. In CNDT 2.5 cells, shRNA mediated knockdown NRP-2 expression led to decreased migration and invasion in vitro (p<0.01). Focused gene-array analysis demonstrated that loss of NRP-2 reduced the expression of a critical metastasis mediator gene, S100A4. Steady-state levels and function of β-catenin, a known regulator of S100A4, were also decreased in the shNRP-2 clones. Furthermore, knockdown of NRP-2 sensitized CNDT 2.5 cells *in vitro* to 5FU toxicity. This effect was associated with activation of caspases 3 and 7, cleavage of PARP, and downregulation of Bcl-2. *In vivo* growth of CNDT 2.5 cells in the livers of nude mice was significantly decreased in the shNRP-2 group (p<0.05). Intraperitoneal administration of NRP-2 siRNA-DOPC decreased the tumor burden in mice (p = 0.01). Collectively, our results demonstrate that tumor cell–derived NRP-2 mediates critical survival signaling in gastrointestinal cancer cells.

## Introduction

Neuropilin-2 (NRP-2) is a transmembrane glycoprotein that was originally described as a receptor for the axon guidance mediators, the semaphorins [1]. Subsequently, it was found to be expressed by venous and lymphatic endothelial cells and identified as a coreceptor for members of the vascular endothelial growth factor (VEGF) family [2], suggesting a role in angiogenesis and lymphangiogenesis [3]. NRP-2 expression has been reported on tumor cells in lung cancer [4], [5], neuroblastoma [6], pancreatic cancer [7], osteosarcoma [8], and bladder cancer [9]. However, the function of the NRP-2 on the tumor cell membrane in human cancers, including those of gastrointestinal (GI) neuroendocrine and gastric origin, remains largely undefined. Previously, we have shown that NRP-2 is expressed on colon and pancreatic cancer cells and that its expression is involved in promoting tumor growth [10], [11]. The purpose of the present study was to determine the function of NRP-2 in mediating downstream signals regulating the growth and survival of human gastrointestinal cancer cells. We found that NRP-2 was overexpressed in human gastric cancer specimens and in gastric and carcinoid cells in vitro. We elucidated the role of NRP-2 in the cell lines with the highest NRP-2 expression. We found that loss of NRP-2 reduced the steady-state levels and function of β-catenin in these cells. NRP-2 knockdown led to decrease in the expression of S100A4 and decreased migration and invasion of the cells in vitro. Furthermore, knockdown of NRP-2 sensitized CNDT 2.5 cells in vitro to 5FU toxicity. These results indicate that NRP-2 mediates critical oncogenic functions in GI cancer cells and that inhibition of its expression and activity could be exploited for therapeutic benefit in patients with metastatic disease.

## Results

### Expression of NRP-2 in human gastric cancer tissue and cell lines

**Figure 1:**
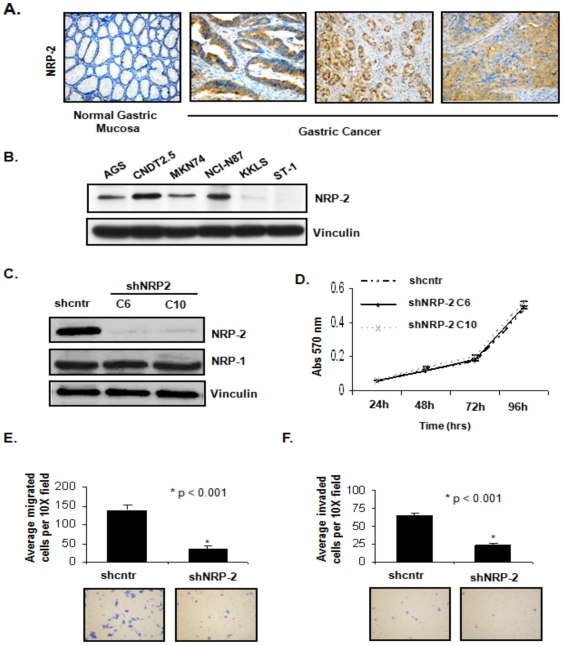
Assessment of NRP-2 expression in human gastric cancer tissues and cell lines. (A) Immunohistochemical staining for NRP-2 expression in representative tissue sections (20X) of normal human gastric mucosa and gastric cancer specimens (B) Immunoblot analysis of NRP-2 expression in six human GI cancer cell lines. Vinculin served as an internal loading control. (C) Generation of stable CNDT 2.5 cell lines with NRP-2 knockdown. Immunoblot analysis of NRP-1 and -2 expression in CNDT 2.5 cells transfected with shcntr or shNRP-2 plasmids (shNRP-2 clones; C6 and C10). Vinculin served as a loading control. (D) MTT assay results. Growth rates were no different between the control cells and NRP-2 knockdown clones. Bars indicate SEM. (E) Top: Mean number of cells that migrated in a Boyden chamber assay. Bottom: Representative images (10X) of migration assays. (F) Top: Mean number of cells that invaded in BioCoat Matrigel invasion chamber assay. Bottom: Representative images (10X) of invasion assays.

We first assessed the expression of NRP-2 protein in paraffin-embedded tissues of human gastric cancer and adjacent normal mucosa by immunoperoxidase staining. In representative gastric cancer specimens, NRP-2 protein was expressed in the gastric cancer epithelium, but not in normal mucosal epithelium (Figure 1A). NRP-2 protein (∼130 kD) was heterogeneously expressed across five of the six gastrointestinal cancer cell lines analyzed by western blotting: AGS, CNDT 2.5, MKN74, NCI-N87 and KKLS (Figure 1B). CNDT2.5 (a human carcinoid cell line [12], [13]) and NCI-N87 expressed the highest levels of NRP-2 and were therefore used for subsequent knockdown studies. As a control, preincubation of the NRP-2 antibody with the immunizing peptide confirmed specificity of the antibody.

### Effect of NRP-2 expression on cell proliferation in vitro

To understand the function of NRP-2 in gastrointestinal cancer, we first examined the effects of NRP-2 silencing on the growth of the CNDT 2.5 cells in vitro. We chose these cells because they express high endogenous levels of NRP-2. CDNT 2.5 cells were stably transfected with a shRNA control (shcntr) or shNRP-2 (shNRP-2) plasmid, and two shNRP-2 transfected clones with a marked decrease in NRP-2 protein expression (C6 and C10, Figure 1C) were selected. shNRP-2 transfection did not affect the expression of NRP-1 in these cells, verifying the specificity of the NRP-2 shRNA. We then used an MTT assay to determine the effect of NRP-2 knockdown on growth rates of the cells in vitro. Clones stably transfected with shNRP-2 showed no change in proliferation rates relative to that of shcntr-transfected cells (Figure 1D).

### Effect of NRP-2 expression on migration and invasion

The effect of NRP-2 RNAi on the motility of CNDT 2.5 cells was examined using Boyden chamber assays. The number of cells that migrated to the bottom chamber was significantly lower in shNRP-2 transfected cells (37±5 per field) than in shcntr transfected cells (140±12 per field) (p<0.001; Figure 1E, top). Conversely, when cells with low endogenous expression of NRP-2 (KKLS) were transiently transfected with a NRP-2 cDNA expression construct in order to overexpress the protein, cell migration was significantly increased (data not shown).

Cell invasion was evaluated using the modified Boyden chamber assay with membranes coated with Matrigel. NRP-2 RNAi significantly inhibited the invasion of CNDT 2.5 cells (64.4%; p<0.001; Figure 1F, top). However, because invasion of cells in this assay is also a function of the migratory potential of the cells, it is difficult to tease out the individual contributions. As such, given that the differences between the two groups is similar in both the assays, we cannot rule out the notion that the reduced number of invading cells may merely reflect decreased migration of shNRP2 clones.

### Effect of NRP-2 knockdown on expression of known metastatic genes

**Figure 2:**
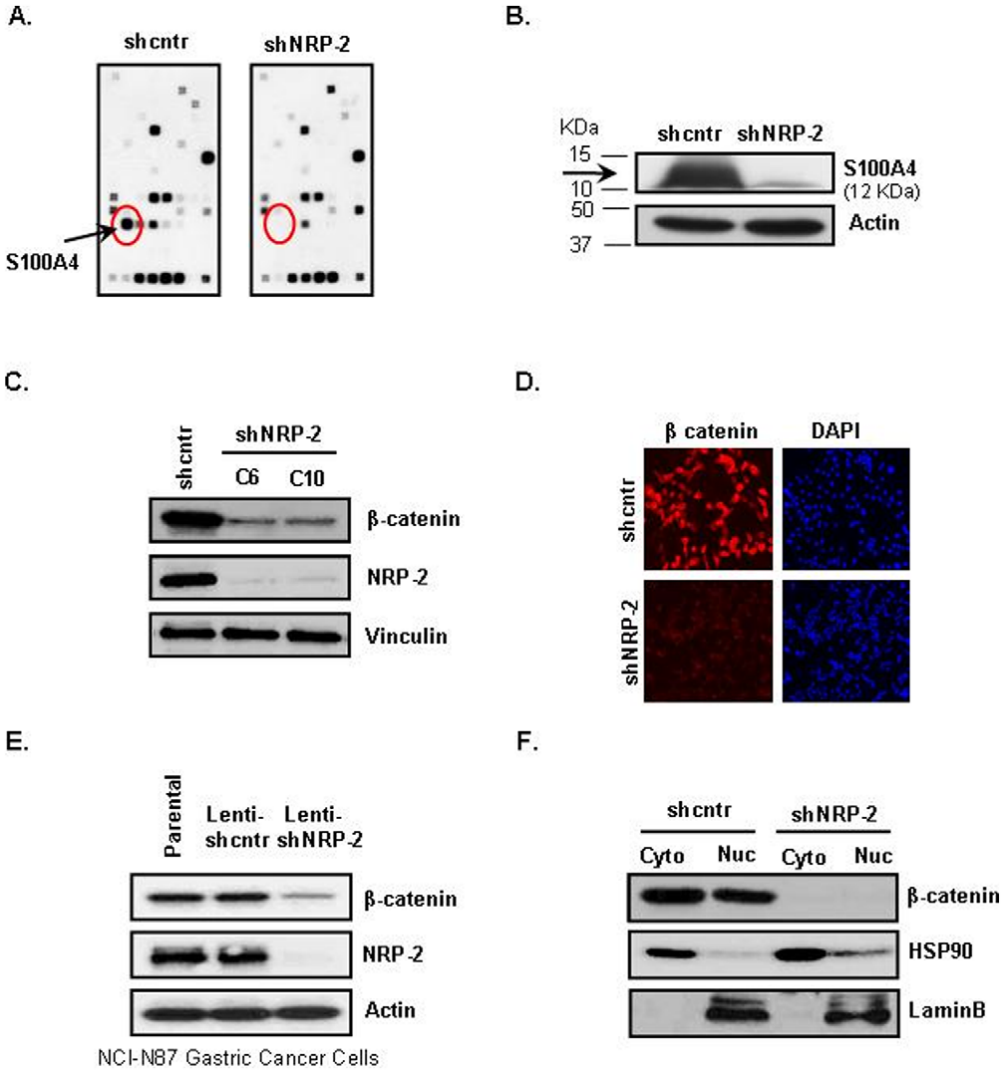
Effect of NRP-2 knockdown on expression of known metastatic genes. (*A*) Autoradiographic image of the array membrane showing differential expression of metastatic genes in shcntr (*left*) and shNRP-2 (*right*) cells. Circled spots indicate the position of the S100A4 gene. (B) Validation of S100A4 protein expression level in cells by immunoblot analysis. Actin served as a loading control. (C) Reduction of the steady-state level of β-catenin by NRP-2 knockdown. β-catenin expression in shcntr and shNRP-2 cells was determined by immunoblotting. Vinculin served as a loading control. (D) Verification of reduced β-catenin expression in shNRP-2 cells by immunofluorescence staining. shcntr and shNRP-2 CNDT 2.5 cells growing in chamber slides were fixed and immunostained with anti-β-catenin antibody (red). Nuclei were counterstained with DAPI (blue). (E) Validation of β-catenin reduction in a second NRP-2 knockdown gastric cancer cell line. NCI-N87 gastric cancer cells were infected with lentivirus containing shcntr or shNRP-2 constructs. Immunoblot analysis showed reduction in β-catenin expression in NCI-N87 NRP-2 knockdown cells. Actin served as a loading control. (F) β-catenin level in the cytoplasmic (Cyto) and nuclear (Nuc) fractions of shcntr and shNRP-2 cells. HSP90 (cytoplasmic) and LaminB (nuclear) served as fractionation controls.

Since invasion and migration are associated with the metastatic phenotype, we performed a gene array analysis using a focused human tumor metastasis microarray that represents 113 genes known to be involved in metastasis. Suppression of NRP-2 caused a reduction in the expression level of several metastatic genes (Figure 2A). Many of the downregulated genes that were responsive to NRP-2 knockdown are known to be associated with degradation of the extracellular matrix and are highly expressed during metastasis. Most notably, we observed that downregulating NRP-2 caused a significant reduction (>95%) in the expression of S100A4 (Figure 2A). Because S100A4 was highly responsive to NRP-2 knockdown, we focused on this gene for further studies. Western blot analysis confirmed that S100A4 protein expression was significantly reduced after NRP-2 knockdown (Figure 2B).

### Effect of NRP-2 knockdown on expression and localization of β-catenin

Because S100A4 is a direct transcriptional target of β-catenin, we examined the expression of β-catenin in the NRP-2 knockdown cells. Total β-catenin protein expression was significantly lower in the CNDT 2.5 shNRP-2 cells than in the shcntr cells (Figure 2C). For further confirmation, shcntr and shNRP-2 clones were fixed and immunostained with anti-β-catenin antibody (red fluorescence). Consistent with the western blot data, total β-catenin levels were significantly reduced in the NRP-2 knockdown cells (Figure 2D). For further validation in a second gastric cancer cell line, NRP-2 was knocked down in NCI-N87 cells, which express a high endogenous level of NRP-2, by lentivirus-mediated stable shNRP-2 incorporation. After verification of NRP-2 knockdown (Figure 2E, Middle panel) in these cells, the β-catenin level was assessed by western blotting. The total β-catenin level was significantly reduced in these NRP-2 knockdown cells (Figure 2E, Top panel).

To determine whether the NRP-2-mediated β-catenin expression was ligand dependent or ligand independent, β-catenin status was evaluated by western blot analysis in CNDT 2.5 cells after treatment with blocking antibodies to NRP-2 (anti-NRP-2^B^, which blocks VEGF-C binding, and pan-anti-NRP, which blocks Sema binding to both NRP-1 and NRP-2 and also blocks VEGF binding through steric hindrance); anti-NRP1 antibody served as a control (all from Genentech, San Francisco, CA). Compared with the control, there was no difference in the expression of β-catenin in cells treated with the blocking antibodies, suggesting that the NRP-2-mediated signaling in these cells is ligand independent (data not shown).

β-catenin is known to exert its function by translocating from the cytoplasm into the nucleus, where it binds to transcription factors such as T cell factor (TCF)/lymphoid enhancer-binding factor and thereby stimulates the transcription of target genes. We therefore analyzed the relative abundance of β-catenin protein in the cytosolic and nuclear compartments in CNDT2.5 shNRP-2 and shcntr cells after cell fractionation. As shown in Figure 2F, NRP-2 knockdown led to significant reduction in the steady-state level of β-catenin protein in both the cytosolic and nuclear fractions of the cells.

### Effect of NRP-2 knockdown on function of β-catenin

**Figure 3:**
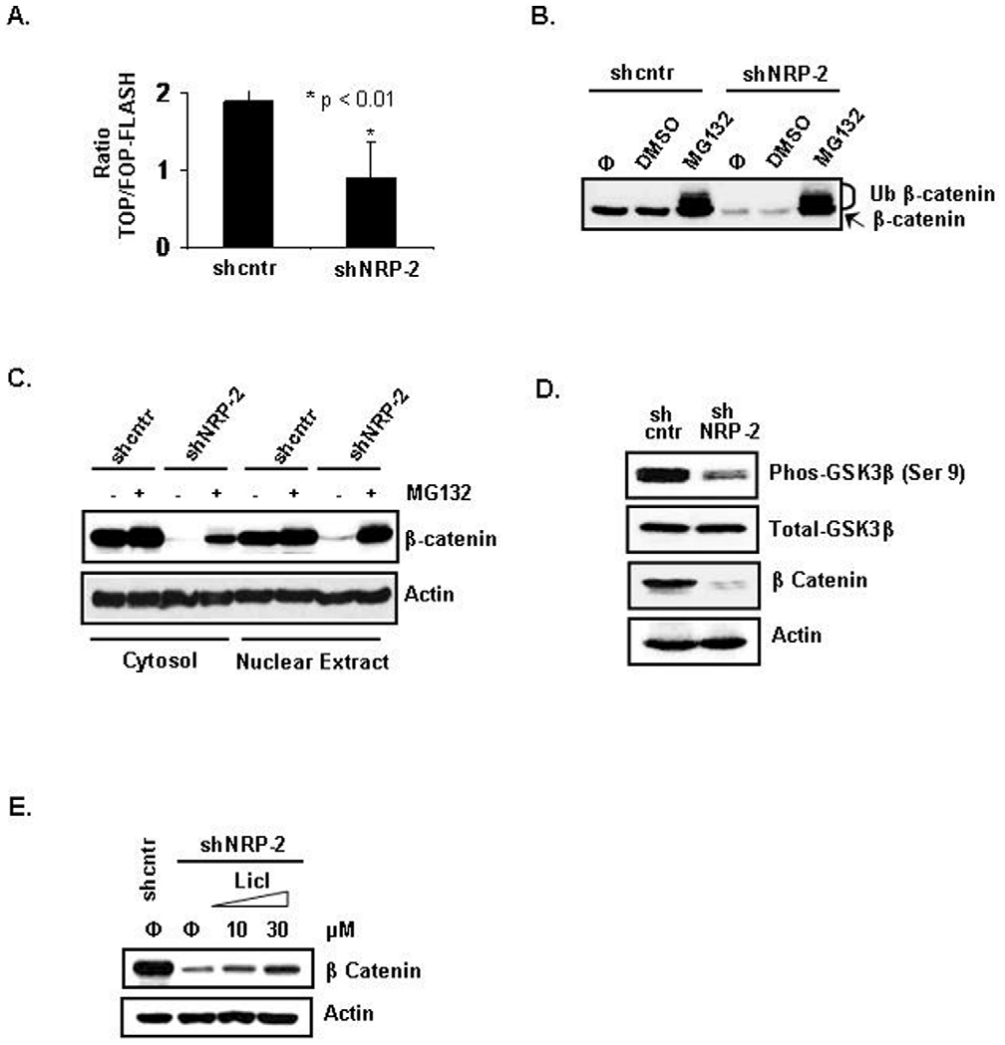
Effect of NRP-2 on β-catenin expression and function. (A) Assessment of TCF reporter activity using the β-catenin-responsive TOPflash or mutant FOPflash reporters. Luciferase activities were measured after transient transfection of the reporter plasmids into shcntr and shNRP-2 CNDT 2.5 cells (B) Increased proteasome-mediated degradation of β-catenin in shNRP-2 cells. shcntr and shNRP-2 CNDT 2.5 cells were treated with 30 µM MG132 or equal volume of DMSO (a solvent for MG132) for 2 hours. Cell lysates were analyzed by immunoblotting using anti-β-catenin antibody (‘φ’, no treatment; ‘Ub’, ubiquitinated β-catenin). (C) Immunoblot analysis showing restoration of β-catenin level in the cytoplasmic and nuclear fractions after treatment with 30 µM MG132 for 2 hours. (D) Decreased levels of phosphorylated GSK3β in NRP-2 knockdown cells. Immunoblot analysis of phosphorylated GSK3β (at Ser-9) and GSK3β. (E) Restoration of β-catenin level after blocking GSK3β activity in NRP-2 knockdown cells. shcntr or shNRP-2 CNDT 2.5 knockdown cells were treated with increasing concentrations of LiCl for 24 hours and harvested for immunoblot analysis to detect β-catenin.

To determine whether NRP-2 knockdown also affected the signaling activity of β-catenin, we transfected CNDT 2.5 shcntr and shNRP-2 cells with the TCF reporter plasmid TOPflash or the control plasmid FOPflash. TOPflash contains a luciferase reporter under the control of three copies of the wild-type TCF-binding element upstream of the thymidine kinase minimal promoter and is specifically regulated by β-catenin signaling. In FOPflash, the TCF-binding element is mutated. The luciferase assay demonstrated that reporter activity was decreased 2-fold in the shNRP-2 cells compared with activity in the shcntr cells (Figure 3A).

### Effect of NRP-2 knockdown on stability of β-catenin

Intracellular β-catenin protein levels are maintained by a multiprotein “destruction complex” that contains APC, Axin, and GSK3β. In an active complex, GSK3β phosphorylates β-catenin, tagging it for recognition by β-trCP, polyubiquitinylation, and subsequent degradation by the proteasome complex. We performed inhibitor studies to assess the stability of β-catenin in the NRP-2 knockdown cells. Treatment with MG132, a proteasome inhibitor, led to restoration of the total β-catenin protein level in the NRP-2 knockdown cells to the level in the shcntr cells (Figure 3B). Furthermore, treatment with MG132 also restored the total β-catenin protein level in both the cytosolic and nuclear compartments in the NRP-2 knockdown cells (Figure 3C).

Posttranslational modifications are known to play a critical role in the regulation of β-catenin turnover, and β-catenin is sequentially phosphorylated by GSK3β. To investigate whether GSK3β activation was involved in the downregulation of β-catenin, we examined the phosphorylation status of GSK3β by use of an antibody against the Ser-9 phosphorylated form of GSK3β. Ser-9 phosphorylation has been reported to render GSK3β inactive. As shown in Figure 3D, Ser-9 phosphorylation of GSK3β was significantly decreased in the shNRP-2 cells, indicating that in these cells there were increased levels of active GSK3β protein, which promotes β-catenin degradation. To further confirm the involvement of GSK3β activation in the regulation of β-catenin, the effect of lithium chloride (LiCl)_,_ a known inhibitor of GSK3β activity, was analyzed. LiCl has been reported to induce the inhibitory Ser-9 phosphorylation of GSK3β while having no effect on other protein kinases. As shown in Figure 3E, treatment with LiCl stabilized β-catenin protein in the shNRP-2 cells in a dose-dependent manner.

### Effect of NRP-2 knockdown on chemosensitivity of gastrointestinal cancer cells in vitro

**Figure 4:**
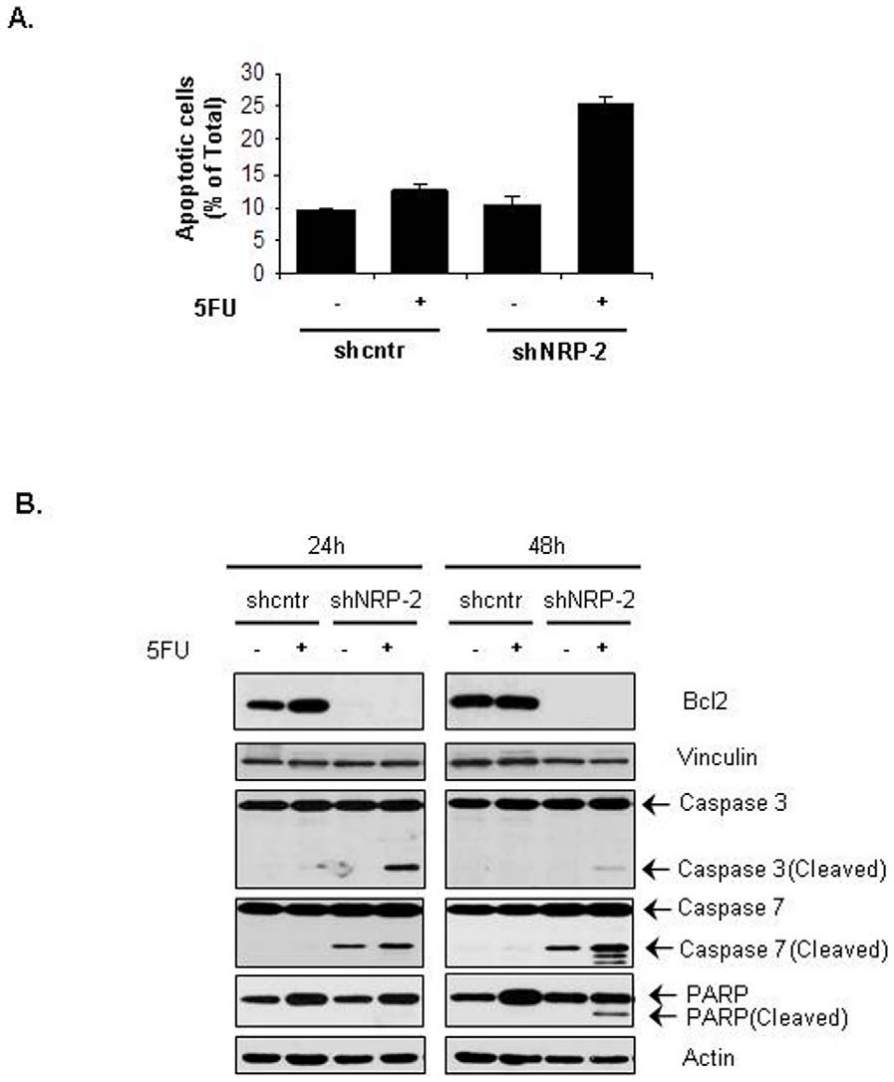
Effect of NRP-2 knockdown on chemosensitivity of CNDT 2.5 cells to 5FU treatment and on expression and activation of apoptotic mediators. (A) Annexin V assay on shcntr and shNRP-2 CNDT 2.5 cells after treatment without or with 5FU for 48 hours. (B) Western blot analysis of apoptotic markers in cell extracts from shcntr and shNRP-2 CNDT 2.5 cells treated without or with 5FU. Vinculin and actin served as loading controls.

Given the known role of β-catenin in mediating cell survival, we hypothesized that a reduced β-catenin level in the NRP-2 knockdown cells would translate into increased chemosensitivity of these cells. To test this hypothesis, we first performed an in vitro chemosensitivity assay with 5-fluorouracil (5FU), which is used as standard therapy for gastrointestinal cancers. Consistent with our hypothesis, by means of annexin V staining, CNDT 2.5 shNRP-2 cells treated with a clinically relevant dose of 5FU demonstrated a significantly higher percentage of apoptotic cells than did shcntr cells (25.5% vs. 12.5%, respectively; p<0.05; Figure 4A).

To confirm the mechanism of 5FU-induced cell death, we compared the activation of apoptotic mediators in the CNDT 2.5 shcntr and shNRP-2 cells treated with 5FU. The activation of caspase-3 and -7, the effector caspases in the apoptotic cascade, and cleavage of the caspase substrate PARP, which exerts proapoptotic activity, were assessed by immunoblotting using antibodies that specifically recognize their cleaved products. Consistent with the increased apoptosis in the 5FU-treated NRP-2 knockdown cells, we observed a marked increase in the levels of active caspase-3 and -7 in these cells, but not in the shcntr cells (Figure 4B). Furthermore, we observed PARP cleavage in 5FU-treated shNRP-2 cells, but not in 5FU-treated shcntr cells (Figure 4B). Additionally, we observed significant expression of the antiapoptotic protein Bcl2 in the CNDT 2.5 shcntr cells; Bcl2 expression was absent in the shNRP-2 cells.

### Effect of NRP-2 knockdown on in vivo growth of gastrointestinal cancer cells

**Figure 5:**
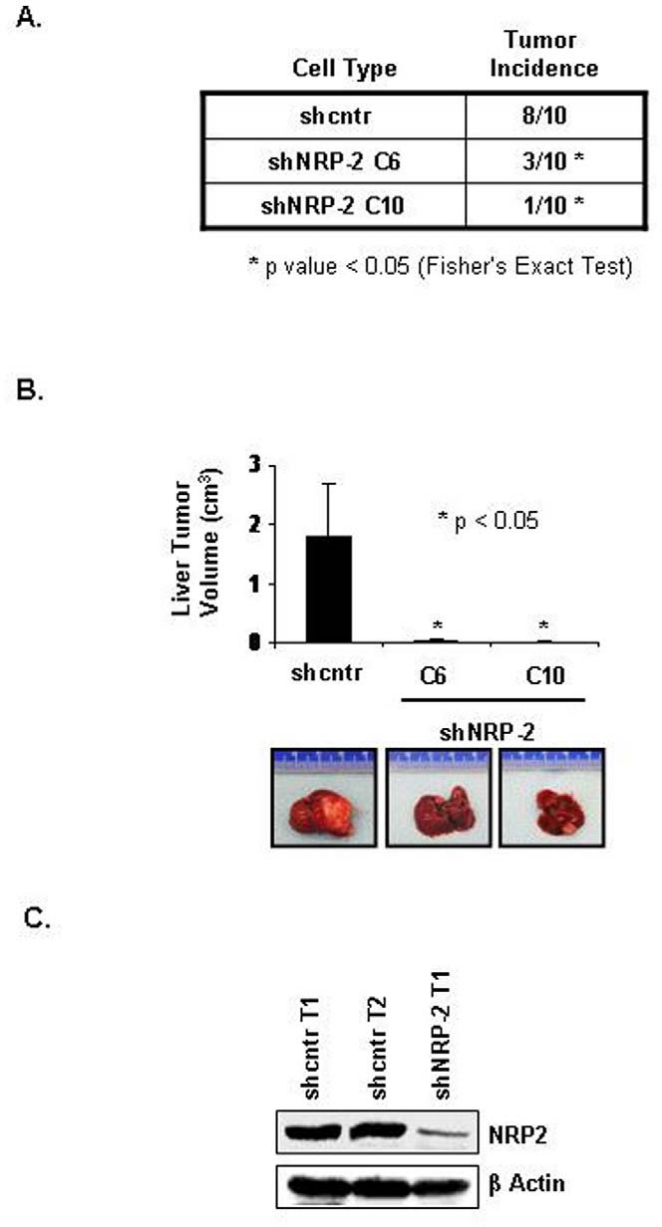
Effect of NRP-2 on in vivo growth of GI cancer cells. (A) Decreased tumor incidence and mean tumor volume after NRP-2 knockdown. Tumor incidence (10 mice) after liver injection with CNDT 2.5 shcntr cells or one of two shNRP-2 clones. (B) Top: Final liver tumor volumes in mice injected with shcntr and shNRP-2 clones. Bottom: Representative images of tumors. (C) Immunoblot analysis of NRP-2 in tumors from mice injected with CNDT 2.5 shcntr or shNRP-2 C6 cells [‘T1’; Tumor #1; ‘T2’; Tumor #2]. β-Actin served as a loading control.

To examine the effect of NRP-2 knockdown on the growth of gastrointestinal cancer cells in vivo, we injected CNDT 2.5 shcntr or shNRP-2 clones in the livers (a common site for gastrointestinal cancer metastasis) of mice (10 animals per group) and assessed tumor incidence and tumor volume. All mice were of approximately the same weight when sacrificed. Tumor incidence was significantly lower in the mice injected with shNRP-2 clones than in the mice injected with shcntr cells (30% for shNRP-2 C6 and 10% for shNRP-2 C10 vs. 80% for shcntr; p<0.05, Fishers Exact Test, Figure 5A). Furthermore, tumors produced by CNDT 2.5 shNRP-2 cells were significantly smaller (Mean liver tumor volume of 38±24 mm^3^ and 14±10 mm^3^ for the two clones, respectively) than were tumors produced by control cells (Mean liver tumor volume of 1,797±880 mm^3^; p<0.05; Figure 5B). The plotted data includes all the 10 mice in each group, including those that did not develop any tumor (ie, 7/10 and 9/10 respectively in the two shNRP2 groups Vs 2/10 in the control group). Representative image of whole liver with tumor from each group is shown in the lower panel. The knockdown of NRP2 in the tumors was confirmed by immunoblot analysis of NRP-2 in liver tumor tissues from mice injected with CNDT 2.5 shcntr or shNRP-2 C6 cells (Figure 5C).

### Effect of in vivo targeting of NRP-2 on growth of gastrointestinal cancer cells

**Figure 6:**
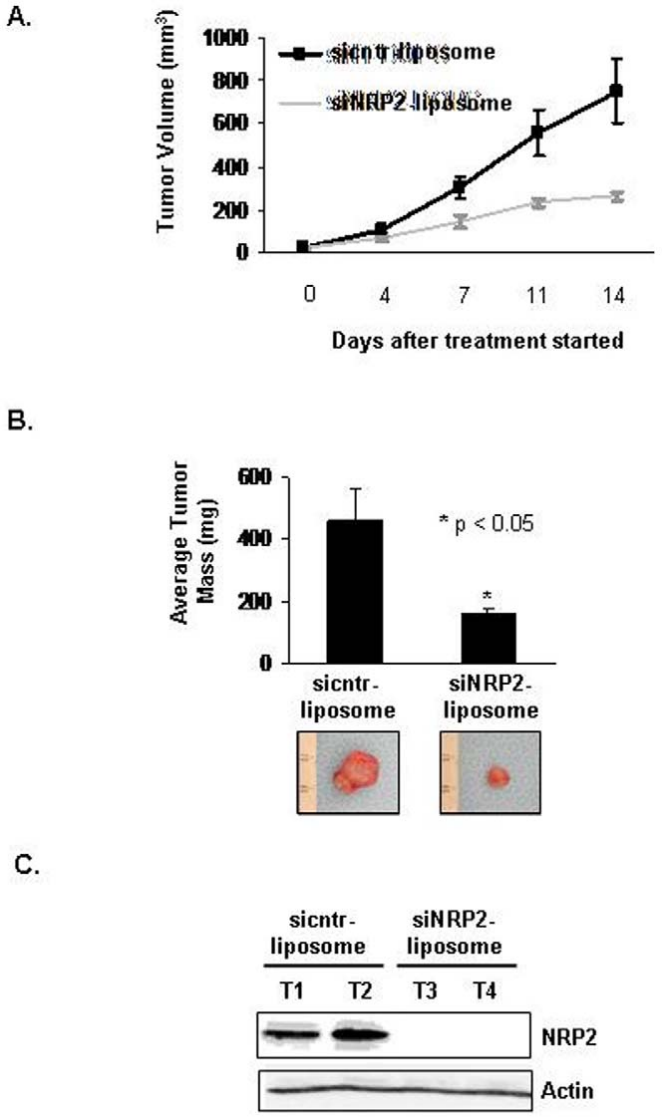
Decreased s.c. growth of CNDT 2.5 cells after systemic administration of DOPC liposome-conjugated siNRP-2. (A) Tumor volumes after administration of sicntr and siNRP-2 liposomes. Nude mice (7 per group) were s.c. injected with 1×10^6^ CNDT 2.5 cells. Mice with visible tumors were stratified and treated with 5 µg of liposome-conjugated siRNA (i.p. injections) twice weekly. NRP-2-siRNA DOPC-treatment led to a significant decrease in tumor growth compared with cntr siRNA DOPC-treatment (264.4 mm^3^ vs. 751.3 mm^3^, respectively; *p = 0.01). (B) Top: At the end of the experiment, s.c. tumors were excised and weighed. Tumor weight was significantly lower in NRP-2-siRNA-DOPC treated mice (mean, 160 mg) compared to cntr siRNA DOPC-treated mice (mean, 459 mg; *p = 0.01). Bottom: Representative images of tumors (C) Immunoblot analysis of NRP-2 in tumors from mice treated with cntr-siRNA DOPC or NRP-2-siRNA DOPC.

Next, we examined the effect of in vivo administration of NRP-2 targeted siRNA using 1,2-dioleoyl-sn-glycero-3-phosphatidylcholine (NRP-2 siRNA-DOPC) neutral nanoliposomes [10], [14] on the growth of gastrointestinal cancer xenografts in mice. CNDT 2.5 cells were grown as s.c. xenografts, and NRP-2 siRNA-DOPC treatment was started on day 7 in mice with visible tumors (15±1.5 mm^3^). Treatment consisted of twice weekly intraperitoneal injection with 5 µg cntr siRNA-DOPC or NRP-2 siRNA-DOPC. The average volume of tumors in mice treated with NRP-2 siRNA-DOPC was less than that for mice treated with cntr siRNA-DOPC sequences (264.4±19.4 mm^3^ vs. 751.3±150.6 mm^3^, respectively; p = 0.01; Figure 6A). Furthermore, mice treated with NRP-2 siRNA-DOPC exhibited a significant reduction in tumor mass compared with tumor mass in mice treated with cntr siRNA-DOPC sequences (160±18 mg vs. 459±102 mg, respectively; p<0.05; Figure 6B). Immunohistochemical staining of tumor tissues confirmed that NRP-2 expression was reduced in the tumors from mice treated with NRP-2 siRNA-DOPC sequences compared with tumors from mice treated with cntr siRNA-DOPC sequences (Figure 6C).

## Discussion

Neuropilin-2 (NRP-2) is traditionally known to function as a nonsignaling coreceptor for class 3 semaphorins and members of the VEGF family [1], [2]. Although the expression of NRP-2 was originally thought to be limited to neurons, studies have shown that NRP-2 is expressed on VSMCs, endothelial cells, and tumor cells. NRP-2 is reported to interact with VEGFR2 and VEGFR3 and increase survival and migration of vascular and lymphatic endothelial cells [15]. Recent functional studies by Caunt and colleagues have shown that blocking NRP-2 in a preclinical lung metastasis model prevented tumor metastasis by blocking the formation of tumor-associated lymphatics [16].

The expression of NRP-2 has been detected on tumor cells of patient samples of many tumor types, including glioblastoma, neuroblastoma, and melanoma. Our laboratory and others have shown that tumor cell–derived NRP-2 plays a role in tumor growth [10], [11]. We sought to further elucidate the functional role of NRP-2 and the signaling mechanisms that mediate the function of NRP-2 in GI cancer cells, including gastric and carcinoid cells. We demonstrate in this study that NRP-2 is highly expressed by tumor cells in gastric carcinoma tissues and by gastrointestinal cancer cell lines, but it is not detectable by immunohistochemistry in normal gastric mucosa. The mechanisms by which NRP-2 levels are upregulated in gastrointestinal cancers remain unclear. Interestingly, Tsukamoto and colleagues [17] found that 40% of gastric cancer patients analyzed had a gain at 2q33 (the region in which the NRP-2 gene is located) by array comparative genomic hybridization (CGH) analysis, suggesting copy number aberration (CNA) of this region.

We utilized a shRNA-mediated RNA interference approach to suppress NRP-2 expression in gastric cancer cells to investigate the resulting phenotypic changes, including proliferative, migratory, and invasive activities. Knockdown of NRP-2 did not affect the proliferation of the CNDT 2.5 cells in vitro; however, loss of NRP-2 reduced the growth of tumor xenografts *in vivo*. The discrepancy between *in vitro* and *in vivo* growth inhibition may be due to NRP-2-mediated responses from cells in the tumor microenvironment at the tumor site *in vivo* that are not appreciable in an *in vitro* system. *In vitro* studies further demonstrated that there was a marked inhibition of both migration and invasion of the cells. Using metastasis-associated genes in a cDNA microarray analysis, we identified S100A4, a key metastasis mediator gene, to be significantly downregulated after NRP-2 silencing. S100A4 has been previously linked to metastasis in several experimental systems [18], [19]. Recent studies have shown that S100A4 may also contribute to cancer progression by enhancing cell survival functions. Mahon and colleagues [20] have demonstrated that S100A4 knockdown sensitizes pancreatic cancer cells to gemcitabine treatment, in addition to activating caspases and PARP, resulting in increased apoptosis and cell cycle arrest. We observed that downregulation of NRP-2 in gastric cancer cells chemosensitized the cells to 5FU treatment. Further studies are needed to determine whether NRP-2 mediates prosurvival functions in gastric cancer cells via S100A4.

Further examination of NRP-2-mediated S100A4 regulation demonstrated that the steady-state levels and function of β-catenin, a direct transcriptional regulator of S100A4, were compromised in the NRP-2 knockdown cells. A large body of data supports the contribution of activation of the β-catenin signaling pathway in the development and progression of cancers. The activation of Wnt/β-catenin signaling is found in about 30% of gastric cancers [21]. In a recent study, Wang and colleagues [22] have proposed β-catenin stabilization as a mode of action for ATDC-mediated oncogenic function in pancreatic cancer. Using a transgenic mouse model, Oshima and colleagues [23] have showed that cooperation of Wnt signaling and the prostaglandin E_2_ (PGE_2_) pathway causes gastric cancer development. The sustained β-catenin pathway activation observed in the NCI-N87 cells must be independent of mutations in the APC and β-catenin genes because this cell line does not harbor any inherent mutations in either of these genes. The mechanism by which NRP-2 silencing affects this pathway remains unclear.

One potential limitation of our study is that we analyzed the growth of NRP-2 knockdown gastric cancer cells as primary tumors but did not evaluate the metastasis of tumors. Given our observation that these cells have decreased migration and invasion after NRP-2 knockdown, the *in vivo* metastatic potential of these cells needs be explored in the future. Further studies are also clearly needed to elucidate the potential mechanism underlying the attenuation of the β-catenin pathway by NRP-2 silencing. This remains a major unresolved issue and is the subject of an ongoing investigation in our laboratory.

NRP-2 is emerging as a novel therapeutic target. Given the role of NRP-2 in tumor cell migration and invasion, targeting NRP-2 offers a potential novel approach to inhibit the growth and survival of cancer cells in patients with metastatic disease. Also, because NRP-2 appears to have roles distinct from that of the VEGF family of receptors, targeting NRP-2 is unlikely to have the same result as targeting VEGF. We have found that treatment of gastrointestinal cancer cells with bevacizumab has no effect on β-catenin expression (data not shown). Targeting NRP-2 may therefore complement and extend the antitumor effects of therapies that target VEGF, such as bevacizumab.

We found that the NRP-2-mediated β-catenin expression observed in this study is ligand independent. This ligand-independent NRP2 signaling is novel however; the mechanism(s) mediating this phenomenon remains to be understood. At the present time, we can only speculate on the mechanism(s) involved, based on those documented for other membrane receptors. (i) It is conceivable that in tumor cells increased expression of NRP2 on the cell surface results in ligand-independent activation of NRP2 mediated signaling through spontaneous NRP2 binding to VEGFR/plexin. (ii) Alternatively, ligand-independent NRP2 signaling could also be activated through homo-dimerization (or heterodimerization with NRP1) as in the case of many other membrane receptors. NRPs are known to form homo- or hetero-multimers even in the absence of ligand through the membrane-proximal c domain. (iii) A third possibility invokes activation via pathway crosstalk with other membrane receptors, but this raises the question of how the postulated receptors are activated. In any instance, proteins that associate with NRP2, including neuropilin interacting protein (NIP)/synectin, are likely to be key to ligand-independent signaling. Based on the ligand-independent nature of NRP2 signalling observed in our study, a therapeutic approach to inhibit NRP2 biological activity in gastric cancer would be using RNA interference.

**Figure 7:**
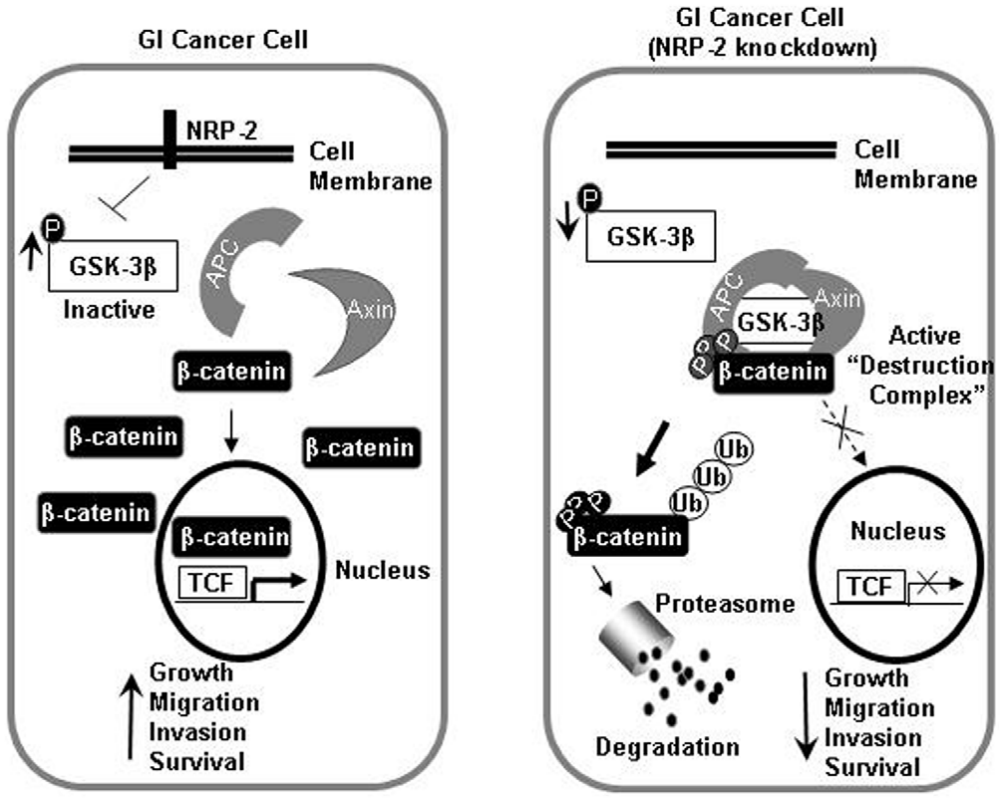
Hypothetical schema for NRP-2-mediated activation of β-catenin signaling in gastrointestinal cancer cells. Left: In a GI cancer cell, increased inactive GSK3β inhibits destruction complex function, leading to stabilization of β-catenin that is free to translocate to the nucleus and activate transcription of target genes. Right: In a GI cancer cell with NRP-2 knockdown, the active destruction complex phosphorylates β-catenin and targets it for proteasome-mediated degradation, resulting in decreased activation of downstream target genes.

The data in this study support the following model for the mechanism by which NRP-2 mediates oncogenic functions in gastrointestinal cancer cells (Figure 7). In gastrointestinal cancer cells, sustained NRP-2 signaling drives inhibition of GSK3β to stabilize β-catenin and engage transcriptional programs that modulate migration, invasion, and survival functions. In summary, our findings implicate NRP-2 as an important positive regulator of β-catenin-dependent signaling in gastrointestinal cancer. NRP-2 mediates critical oncogenic functions in gastrointestinal cancer cells and therefore is a potential therapeutic target for the disease.

## Materials and Methods

### Human tissue specimens

Formalin-fixed, paraffin-embedded specimens of gastric cancer tissues and adjacent non-malignant gastric mucosa were obtained from The University of Texas MD Anderson Cancer Center tissue bank through a protocol approved by the institutional review board of The University of Texas MD Anderson Cancer Center, Houston, TX, USA. The specimens were collected retrospectively and therefore a waiver was granted by our Institutional Review Board. Histopathologic confirmation was provided by the MD Anderson Cancer Center's Department of Pathology.

### Histopathologic preparation and immunohistochemical staining of tissue specimens

Patient tissue specimens were stained with an anti-NRP-2 antibody (Santa Cruz Biotechnology, Santa Cruz, CA) as described previously [10]. As a control, preincubation of the NRP-2 antibody with the immunizing peptide confirmed specificity of the antibody.

### Cell lines and cell culture conditions

NCI-N87 and AGS cells were obtained from the ATCC (Manassas, VA). MKN74 cells were a kind gift from Dr. Gary Schwartz (Memorial Sloan-Kettering Cancer Center, New York, NY). CNDT 2.5 cells were established previously in our laboratory from liver metastases of a chemonaïve patient with a history of primary ileal carcinoid tumor using an algorithm developed by our laboratory [12]. KKLS cells were obtained from Dr. Yukuta Takahashi (Cancer Research Institute, Kanazawa University, Kanazawa, Japan). ST-1 cells (a gift from Dr. Bradley McIntyre) were established from a gastric cancer patient at M. D. Anderson Cancer Center. All cells were maintained in complete DMEM supplemented with 10% FBS, 2 mM L-glutamine, and 100 units/ml penicillin and streptomycin (Life Technologies, Grand Island, NY) at 37°C in a humidified atmosphere containing 5% CO_2_.

### Construction of shRNA targeting NRP-2 and generation of stable shRNA cell lines

shcntr and shNRP-2 expression plasmids were created as described previously [10]. Briefly, two pairs of shRNA were designed using the Ambion siRNA design tool (www.Ambion.com) and introduced into the pSilencer 4.0 vector (Ambion, Austin, TX) according to the manufacturer's directions. The negative control consisted of a pSilencer 4.0 plasmid containing scrambled NRP-2 target sequences (5′-agatcggtggcctatagaacg and 5′-gatcatcaccttggaccagac). CNDT 2.5 cells were transfected with 0.5 ng of each shRNA plasmid as previously described [10] and grown in selective medium containing 850 µg/ml hygromycin B (Roche Diagnostics, Mannheim, Germany).

### MTT analysis of cell proliferation

In vitro proliferation was analyzed with the reducing tetrazolium salt 3-(4,5-dimethylthiazol-2-yl)-2,5-diphenyltetrazolium bromide (MTT) assay. Briefly, 2,000 cells of each clone were plated per well onto 96-well microtiter plates in DMEM with 10% FBS. Assays were performed by adding 20 µl of MTT substrate to each well for 2 hours, followed by removal of media and the addition of 200 µl DMSO. Spectrometric absorbance was measured on a microplate reader (Bio-Rad, Hercules, CA) at a wavelength of 570 nm.

### In vitro migration and invasion assays

The inhibitory effect of RNAi on migration of CNDT 2.5 cells in vitro was assayed using modified Boyden chambers (Coster, Boston, MA). shcntr or shNRP-2 cells (1×10^5^) were suspended in 500 µl DMEM with 1% FBS and added to the upper chamber. DMEM (750 µl) with 10% FBS was added to the bottom chamber. The chambers were incubated for 48 hours at 37°C in a humid atmosphere of 5% CO_2_. After incubation, the upper surface of the filters was scraped twice with cotton swabs to remove cells. The filters were fixed and stained with Diff-Quick reagent (Dade Behring, Newark, DE). For the invasion assay, the procedure was similar to that for the migration assay except that a BioCoat Matrigel invasion chamber (Becton Dickinson, Bedford, MA) was used. All experiments were repeated in triplicate wells, and the numbers of invading cells in five high-power fields per filter were counted at x10 magnification.

### Nuclear fractionation, preparation of whole-cell lysates and western blot analyses

Nuclear proteins were extracted using the Nuclear Extract Kit (Active Motif, Carlsbad, CA) according to the manufacturer's instructions. Whole-cell lysates were obtained by incubating cells in RIPA solution containing protease inhibitors (Roche Diagnostics, Nutley, NJ). Protein concentrations were determined using a protein assay kit (Bio-Rad, Hercules, CA). Western blotting was performed with 50 µg of total cellular protein from each cell line. Proteins were separated on a 10% SDS–PAGE, transferred onto a PVDF membrane (Amersham Biosciences, Arlington Heights, IL), and probed with antibodies against NRP-1 and NRP-2 (Santa Cruz Biotechnology, Santa Cruz, CA); β-catenin (Upstate/Millipore, Billerica, MA); caspase 3, caspase 7 and PARP and their cleaved forms (Cell Signaling Technology, Danvers, MA). Blots were incubated with HRP–conjugated secondary antibodies, and proteins were visualized using ECL reagent (Amersham Biosciences, Arlington Heights, IL). Antibodies used in the blocking experiment included anti-NRP-2^B^, pan-anti-NRP, and anti-NRP1 (all from Genentech, San Francisco, CA).

### cDNA microarray analysis

Total RNA from shcntr and shNRP-2 cells was extracted using Trizol reagent (Invitrogen, Carlsbad, CA). cDNA was prepared from 5 µg of total RNA and labeled in a RT reaction with biotin-16-dUTP (Roche). The labeled cDNA was hybridized to the Oligo GEArray Human Tumor Metastasis Microarray (Cat #OHS-028; SABiosciences, Frederick, MD) and developed as per manufacturer's instructions. The results were analyzed by GEArray Analyzer software (SABiosciences).

### Lentiviral stable shRNA cell lines

Lentiviral vectors (Open Biosystems, Huntsville, AL) containing control or NRP-2 shRNA were generated as per manufacturers instructions and used to infect NCI-N87 gastric cancer cells.

### β-Catenin luciferase reporter assay

CNDT 2.5 cells were transiently transfected with 1 µg of either TOPflash or FOPflash TCF reporter constructs (Millipore, Billerica, MA) using Lipofectamine 2000 (Invitrogen, Carlsbad, CA), and luciferase activity was measured 48 hours after transfection. Luciferase assays were performed with a dual luciferase assay kit as per the manufacturer's instructions (Promega, Madison, WI). TCF activity was calculated in terms of extent (fold) of change in activity (TOPflash/FOPflash).

### Preparation of siRNA-containing liposomes

Liposomes containing cntr and NRP-2 targeted siRNA sequences were prepared as previously described [10]. Briefly, DOPC and siRNA were mixed and lyophilized. The lyophilized preparations were hydrated in PBS at a concentration of 5 µg/100 µl before in vivo administration. DOPC nanoliposomes were detremined to have a mean diameter of 30 nM.

### In vivo tumor growth studies

Male athymic nude mice (6–8 weeks) were purchased from the NCI (Frederick, MD) and were maintained under specific pathogen-free conditions at the M. D. Anderson Cancer Center Animal Care Facility and their use in these experiments was approved by the IACUC (#04-07-03032). shcntr, shNRP-2 C6, and shNRP-2 C10 cells (1×10^6^ cells per injection in 0.05 ml HBSS) were injected directly into the liver of each anesthetized mouse (Ten mice per clone). Mice were sacrificed at 21 days. Tumor volumes were calculated as [(length) × (width^2^/2)].

For the systemic siRNA delivery experiment, 1×10^6^ CNDT 2.5 cells (in 0.1 ml HBSS) were injected s.c. into each mouse (right rear flank). Mice with visible tumors were stratified and administered cntr-siRNA DOPC or NRP-2-siRNA DOPC complexes (5 µg siRNA per injection) as a 100 µl bolus i.p. twice weekly. After 5 treatments, the mice were sacrificed, and tumors were harvested and analyzed.

### Annexin V staining

The percentage of apoptotic cells was assessed using the Annexin V FITC Apoptosis Detection Kit (BD Pharmingen, San Diego, CA) according to the manufacturer's instructions. Annexin V quantitation was performed using a Coulter EPICS XL-MCL FAC analyzer (Beckman Coulter, Miami, FL).

### Statistical analyses

All data are expressed as mean ± SE. Statistical analyses were performed with InStat 2.01 statistical software (GraphPad Software, San Diego, CA). The differences between groups were determined by paired Student *t*-test, Fisher exact test or Mann-Whitney U tests. p values <0.05 were considered statistically significant.
